# Late-onset diffuse lamellar keratitis 4 years after femtosecond laser-assisted small incision lenticule extraction: a case report

**DOI:** 10.1186/s12886-017-0641-x

**Published:** 2017-12-08

**Authors:** Meiyan Li, Dong Yang, Yingjun Chen, Meng Li, Tian Han, Xingtao Zhou, Katherine Ni

**Affiliations:** 1 Key Lab of Myopia, Ministry of Health, Department of Ophthalmology, EYE & ENT Hospital of Fudan University, Shanghai, China; 2 School of Medicine, New York University, New York, USA

**Keywords:** Late-onset, DLK, Smile, Trauma

## Abstract

**Background:**

To report a first case of late-onset diffuse lamellar keratitis (DLK) occurring 4 years after femtosecond laser-assisted small incision lenticule extraction (SMILE).

**Case presentation:**

A 41-year-old man who underwent SMILE 4 years prior developed DLK in the right eye 1 day after he was struck in the eye by a finger while playing with his son. Slim-lamp microscopy and anterior segment optical coherence tomography (AS-OCT) were used to evaluate the cornea of the right eye. Slit-lamp examination of the right eye revealed epithelial exfoliation and stage 3 DLK with diffuse, dot-like, granular haze in the interface between the cap and stromal bed. After intensive treatment with topical corticosteroids, the DLK resolved and corneal transparency was achieved.

**Conclusions:**

This case indicates that DLK can occur several years after SMILE. Ocular trauma may be a risk factor for the development of DLK. The prognosis is usually favorable with early diagnosis and treatment with topical corticosteroids.

## Background

Diffuse lamellar keratitis (DLK) is a condition in which a white blood cell infiltrate accumulates between the flap and stromal bed, and is typically a potential early complication after laser in situ keratomileusis (LASIK) [1, 2]. It has also been reported with an incidence of 1 in 62 after small incision lenticule extraction (SMILE) [3]. Late-onset DLK has been described in several studies after LASIK [4, 5], however most of these cases are likely due to a specific causative agent such as trauma or epithelial defects [6]. As far as we know, no paper about late-onset DLK after SMILE has yet been described in the scientific literature. In this case report, we present a patient with late-onset DLK, induced by trauma occurring 4 years after SMILE.

## Case presentation

A 41-year-old man had undergone femtosecond laser-assisted SMILE in the right eye on November 1, 2011 at the Department of Ophthalmology of Fudan University Eye and ENT Hospital (Shanghai, People’s Republic of China). Preoperatively, refraction was −8.75/ -1.00 × 35 in the right eye and his corrected distance visual acuity (CDVA) was 20/25. Keratometric readings (Pentacam; Oculus, Wetzlar, Germany) were 44.70/44.90 diopters. Central corneal thickness was 565 μm. No anterior and posterior segment abnormalities were observed. The femtosecond laser system (VisuMax, Carl Zeiss Meditec AG, Jena, Germany) was used to perform the SMILE procedure. The repetition rate was set to 500 kHz, with a pulse energy of 180 nJ. The surgery was conducted uneventfully, as described in our previous report [7]. One day postoperatively, the uncorrected distance visual acuity (UDVA) was 20/50. At 6 months, it was 20/25 and the CDVA was 20/25 with a manifest refraction of +0.25 /−0.75 × 15.

**Fig. 1:**
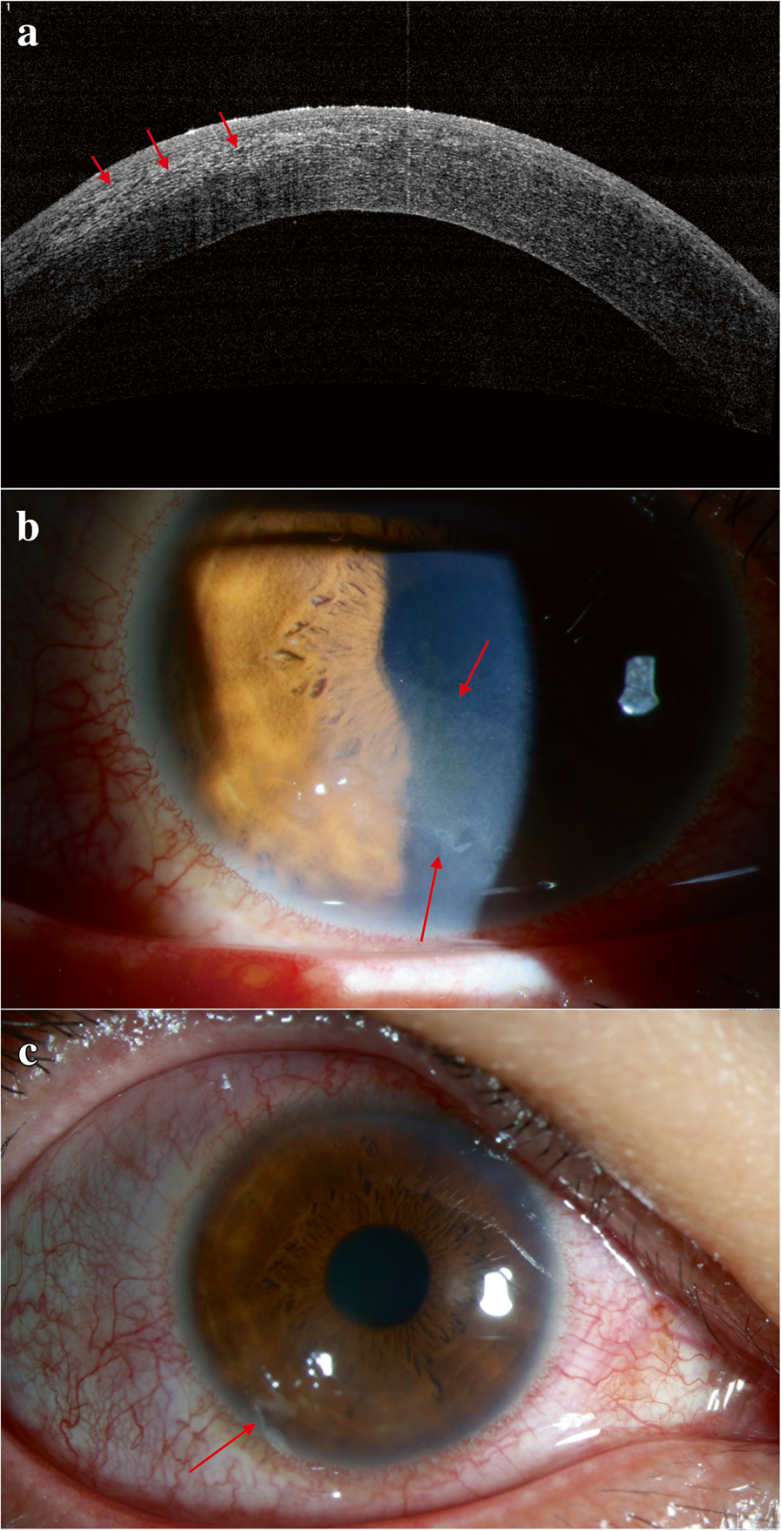
The anterior segment optical coherence tomography (AS-OCT) showing (**a**) hyper reflection in the left corneal stromal bed (red arrow); slit-lamp photography showing diffuse, dot-like, and granular haze in the interface between cap and stromal bed (red arrow) (**b**) and epithelial exfoliation (red arrow), conjunctiva edema and hyperaemia of the right eye (**c**) at 1 day after trauma (58 months after SMILE)

**Fig. 2:**
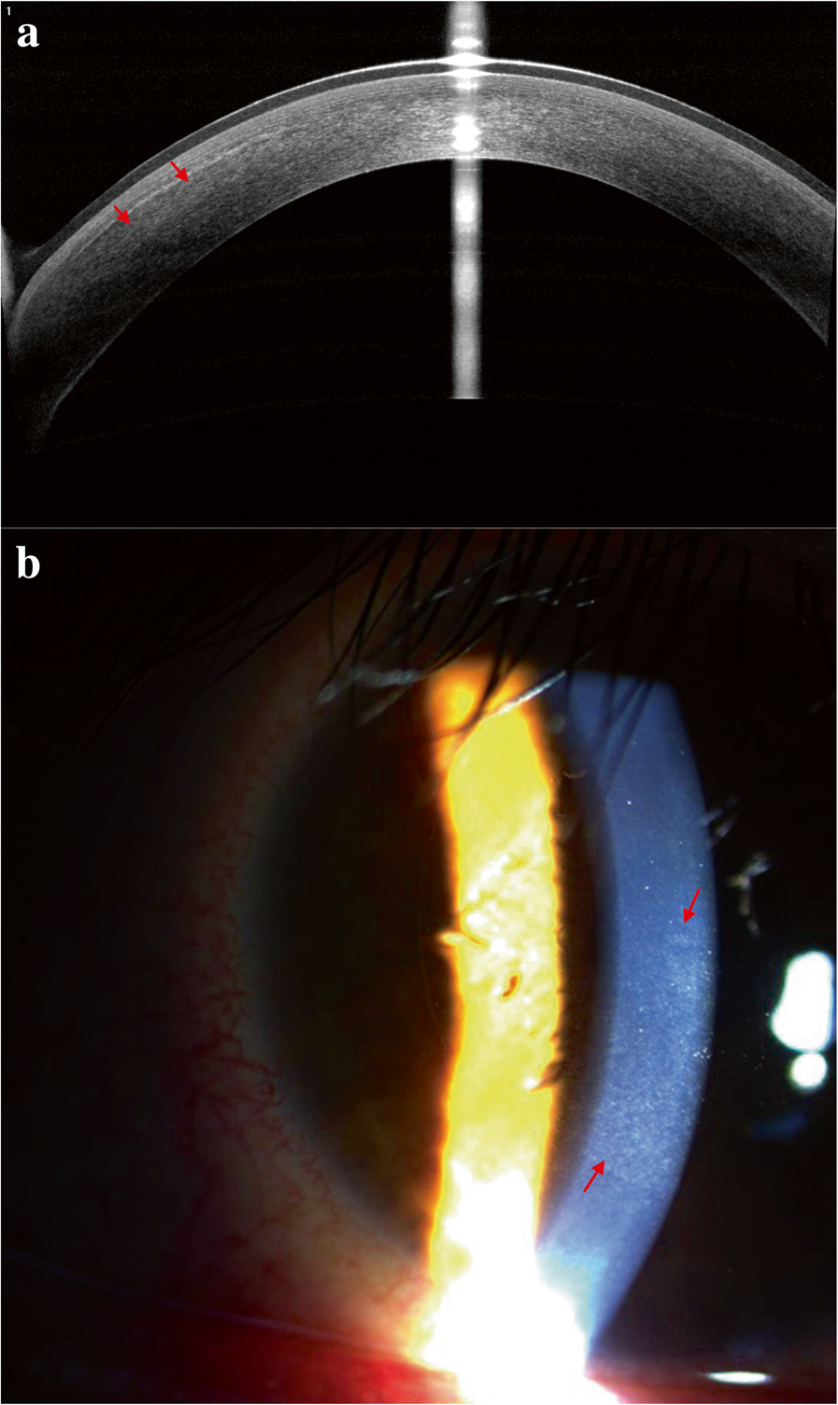
The anterior segment optical coherence tomography (AS-OCT) showed (**a**) hyper reflection has reduced in the left corneal stromal bed (red arrow); slit-lamp photography showed diffuse, dot-like, and granular haze has relieved in the interface between cap and stromal bed (red arrow) (**b**) at 5 day after treatment

**Fig. 3:**
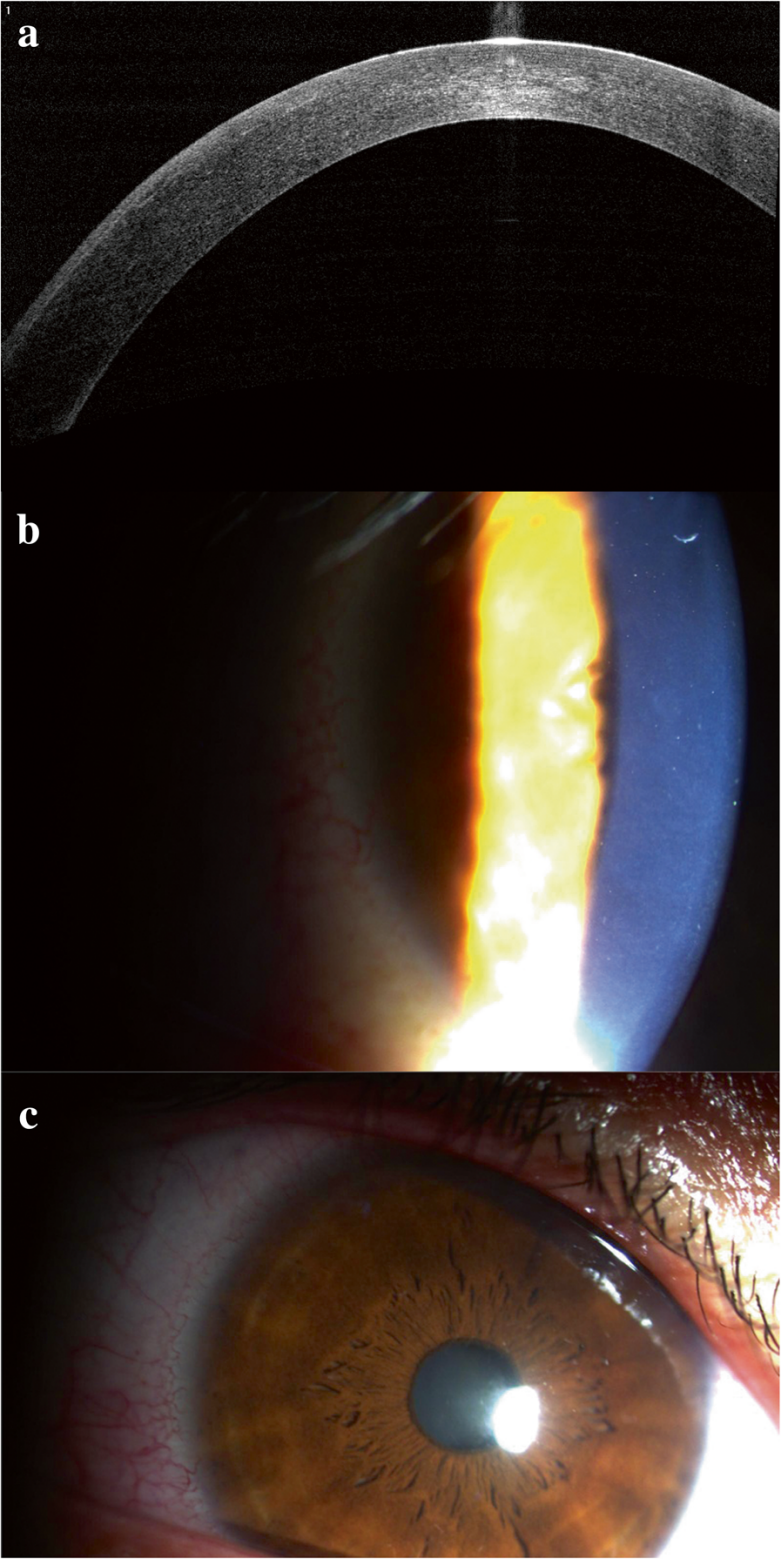
The AS-OCT (**a**) and slit-lamp (**b** and **c**) images of the right eye showed resolution of diffuse lamellar keratitis after corticosteroid treatment, at 10 days after trauma (58 months after SMILE)

In August 2016, 58 months after the SMILE surgery, the patient noted a decline in visual acuity, accompanied by sharp jabbing pain in the right eye, starting 1 day after being struck in the right eye with a finger while playing with his young son. He presented to our hospital, where his UDVA was found to be 20/200 OD. Slit-lamp examination of the right eye revealed epithelial exfoliation and stage 3 DLK with a diffuse, dot-like, granular haze in the interface between the cap and stromal bed. Anterior segment optical coherence tomography (AS-OCT) and slit-lamp findings at presentation are shown in Fig. 1. Bandage soft contact lens (ACUVE OASYS, Inc., FL, USA) was applied to the right eye. Prednisolone acetate 1.0% was prescribed 8 times daily and tapered every day for the first three days, then 5 times daily for two days, along with levofloxacin eyedrops 4 times daily. Five days after initiating treatment, the pain has relieved and the UDVA increased to 20/80, the area of DLK was smaller and the epithelial defect was healed (Fig. 2). The bandage soft contact lens was discontinued. Prednisolone acetate 1.0% was prescribed 4 times daily and tapered every 2 days. Ten days after initiating treatment, the clinical signs of DLK had resolved. AS-OCT and slit-lamp images at day 10 are shown in Fig. 3. The UDVA returned to 20/25 and the CDVA was 20/25 with a manifest refraction of +0.25 /−0.75 × 20. Two weeks after the injury, the patient’s UDVA returned to 20/25 in the right eye, and the prednisolone acetate was discontinued.

## Discussion and conclusions

Several reports have described cases of late-onset DLK induced by trauma after LASIK with flap displacement [8, 9]. The majority of the reported cases, which occurred between 1 and 12 months postoperatively, were associated with a traumatic or spontaneous epithelial defect [10, 11]. Haw et al. have reported that epithelial injury could induce alterations in the metabolism and oxygenation of the cornea and allow more diffusion of inflammatory mediators from the tear film or alter the permeability of the limbal vasculature to inflammatory cells [10]. Our case illustrated the development of late-onset DLK 58 months after SMILE, induced by trauma in the eye. As SMILE leaves the anterior cornea intact, no flap displacement occurred due to the trauma.

Rana et al. [12] reported that late-onset stage 3 DLK after LASIK should be managed by lifting of the flaps and performing an interface washout, if the response to treatment with intensive topical steroids is poor. Zhao et al. [3] have described that with timely diagnosis and topical steroid administration, the prognosis for DLK occurring within a few days after SMILE is usually good and the refractive outcomes (UDVA, CDVA, and manifest refraction) are comparable to those without DLK, even in patients with stage 3 DLK. In our study, we report a case of late-onset stage 3 DLK after SMILE, which, like early-onset DLK after SMILE, responded well to treatment with topical corticosteroids.

Our study indicates that DLK may occur several years after SMILE, and may be induced by trauma. Early diagnosis and treatment is important for the prognosis of late-onset DLK, and the same principles of treatment may be applied to both late-onset and early-onset DLK after SMILE and LASIK.

## Abbreviations

AS-OCT: Anterior segment optical coherence tomography
CDVA: Corrected distance visual acuity
DLK: Diffuse lamellar keratitis
LASIK: Laser in situ keratomileusis
SMILE: Small incision lenticule extraction
UDVA: Uncorrected distance visual acuity
